# The performance of MR perfusion-weighted imaging for the differentiation of high-grade glioma from primary central nervous system lymphoma: A systematic review and meta-analysis

**DOI:** 10.1371/journal.pone.0173430

**Published:** 2017-03-16

**Authors:** Weilin Xu, Qun Wang, Anwen Shao, Bainan Xu, Jianmin Zhang

**Affiliations:** 1 Department of Neurosurgery, Second Affiliated Hospital, School of Medicine, Zhejiang University, Hangzhou, Zhejiang, China; 2 Department of Neurosurgery, Chinese PLA General Hospital, Haidian District, Beijing, China; 3 Brain Research Institute, Zhejiang University, Hangzhou, Zhejiang, China; 4 Collaborative Innovation Center for Brain Science, Zhejiang University, Hangzhou, Zhejiang, China; George Washington University, UNITED STATES

## Abstract

It is always a great challenge to distinguish high-grade glioma (HGG) from primary central nervous system lymphoma (PCNSL). We conducted a meta-analysis to assess the performance of MR perfusion-weighted imaging (PWI) in differentiating HGG from PCNSL. The heterogeneity and threshold effect were evaluated, and the sensitivity (SEN), specificity (SPE) and areas under summary receiver operating characteristic curve (SROC) were calculated. Fourteen studies with a total of 598 participants were included in this meta-analysis. The results indicated that PWI had a high level of accuracy (area under the curve (AUC) = 0.9415) for differentiating HGG from PCNSL by using the best parameter from each study. The dynamic susceptibility-contrast (DSC) technique might be an optimal index for distinguishing HGGs from PCNSLs (AUC = 0.9812). Furthermore, the DSC had the best sensitivity 0.963 (95%CI: 0.924, 0.986), whereas the arterial spin-labeling (ASL) displayed the best specificity 0.896 (95% CI: 0.781, 0.963) among those techniques. However, the variability of the optimal thresholds from the included studies suggests that further evaluation and standardization are needed before the techniques can be extensively clinically used.

## Introduction

Gliomas are the most common type of primary neoplasms in adults [1]. Patients who are afflicted with glioma, particularly high-grade glioma (HGG), always have a short lifespan and poor quality of life. In general, the HGGs were more likely to be rim-like lesions on the MR imaging while the PCNSLs were more likely to be homogeneous enhancing masses. However, in many cases, conventional MR imaging of primary central nervous system lymphoma mimics that of the high-grade glioma, which could all appear with rim-like enhancement with necrosis or could manifest as homogeneous enhancing masses [2–3]. However, the treatment strategies are completely different. Therefore, accurately differentiating HGG from PCNSL is quite important for the adoption of eligible treatment strategies to minimize the risk for those patients [4–6].

Given the limitations of conventional MRI in differentiating HGG from PCNSL, an increasing number of studies have recently focused on monitoring the physiological and metabolic characteristics of tumors [2,3,7,8]. MR perfusion imaging, including the dynamic susceptibility-contrast (DSC)-MRI, dynamic contrast-enhanced (DCE)-MRI, intra-voxel incoherent motion (IVIM)-MRI and arterial spin-labeling (ASL)-MRI techniques, could provide information about the micro-vascular physiology of tumors. Among the techniques of MR perfusion imaging, DSC is the most widely used. The main application of DSC is to quantitatively detect the cerebral blood volume (CBV) in different lesions [7]. Compared with the DSC technique, IVIM has the advantage of providing quantitative measurements of both the tumor cellularity and vascularity [9]. ASL is an emerging MR perfusion imaging technique that requires no extrinsic tracer or radiation exposure, which is a benefit of ASL over other perfusion imaging techniques [10]. Additionally, DCE has the ability to obtain characteristics of the vascular microenvironment such as vascular permeability [8].

It has been reported that HGG and PCNSL share different vascularity features [7,8,11]. Therefore, PWI holds promise in separating HGG from PCNSL on the basis of their different characteristics of angiogenesis and neovascularity [11–14]. However, individual studies have used different techniques on heterogeneous patient groups and included a small number of cases, thus making it difficult to systematically evaluate the performance of PWI. Therefore, we perform this meta-analysis systematically to assess the diagnostic accuracy of MR perfusion in distinguishing HGG from PCNSL based on the eligible published studies.

## Materials and methods

### 2.1. Search strategy

We conducted this meta-analysis according to the PRISMA guidelines (S1 Table). A systematic literature search was conducted in Embase, PubMed, and Chinese Biomedical databases to select eligible studies by using a combination of free-text words and MeSH terms as follows: (perfusion/PWI/perfusion weighted imaging/magnetic resonance perfusion/MR perfusion/perfusion image) AND (glioma/brain neoplasm/brain tumor) AND (lymphoma). The search time was from the database inception to October 1, 2016, with the language restricted to English and Chinese. The reference lists of all eligible studies were hand-searched for underlying relevant articles.

### 2.2. Selection criteria

The inclusion criteria were as follows: (1) the study utilized PWI techniques to distinguish PCNSLs from HGGs, and the patients included had no pre-surgical adjuvant treatments; (2) the reference standard was pathological diagnosis, and the numbers of PCNSLs and HGGs could be obtained; (3) at least one parameter was used to differentiate HGGs from PCNSLs; (4) the sensitivity and specificity could be calculated from the data; (5) at least 8 patients were included in each study; (6) there were no overlapping data; and (7) there were only English and Chinese articles with full-text publications. The following types of studies were excluded: reviews, letters, editorials, abstracts, case reports, proceedings, and personal communications.

The data from the potentially eligible studies were extracted and summarized individually by two of the reviewers (W.l. Xu and Q. Wang). Any disagreement was settled by a third reviewer (J.M. Zhang).

### 2.3. Data extraction and quality assessment

The last process to evaluate the articles included was completed individually by two of the reviewers (A.W. Shao and W.l. Xu). The following basal characteristics were obtained: authors, years, country, study design, number of patients included in each study, age and gender, pathology, reference standards and technical information (strength of image field, technique of PWI, parameters, cut-off value).

For the differentiation, HGGs (grades Ⅲ-Ⅳ) were positive, and PCNSLs were negative. The TP, FP, FN and TN values from each study were calculated. Two of the authors independently assessed the methodological quality of the studies using the Quality Assessment Tool for Diagnostic Accuracy Studies version 2 (QUADAS-2) [15]. Any discrepancies were resolved by an adjudicating senior author.

### 2.4. Statistical analysis

We used standard methods to evaluate the diagnostic accuracy [16–17].

First, we evaluated the threshold effect by adopting the Spearman correlation coefficient between the logit of SEN and the logit of (1−S; first, the heterogeneity was evaluated between each study that may have been caused by PE). A threshold effect existed if the value of P < 0.05.

Then, a chi-squared value test and inconsistency index (I2) of the diagnostic odds ratio (DOR) were used to assess the heterogeneity in each study. If severe heterogeneity was present with a value of P < 0.1 or I2> 50%, the random effect models were chosen; otherwise, the fixed effect models were used. We performed meta-regression analyses to find the source of heterogeneity [18,19].

We calculated the pooled sensitivity, specificity, LR+, LR−, and diagnostic odds ratios (DOR) with their 95% confidence intervals (CIs) with the best performing parameter from each study, and the same principle was used in the subgroup analyses. We added a value of 0.5 to all cells of studies that had SENs or SPEs of 100%. We calculated the SROC, AUC and Q* index (i.e., the point on the SROC at which SEN and SPE are equal; this is the best statistical method for assessing diagnostic performance). AUC values ranging from 51% to 70%, from 71% to 90%, and >90% suggested low, moderate, and diagnostic accuracy, respectively. The minimum number of studies required to form a subgroup was 3. The statistical analyses mentioned above were conducted using the Meta-DiSc statistical software version 1.4 [17].

Publication bias was assessed by Deek’s funnel plot. Formal testing for publication bias was conducted with P < 0.1 showing significant asymmetry [20]. This process was conducted using Stata14.0 (StataCorp LP, College Station, TX).

## Results

### 3.1. Literature search and study characteristics

**Fig 1:**
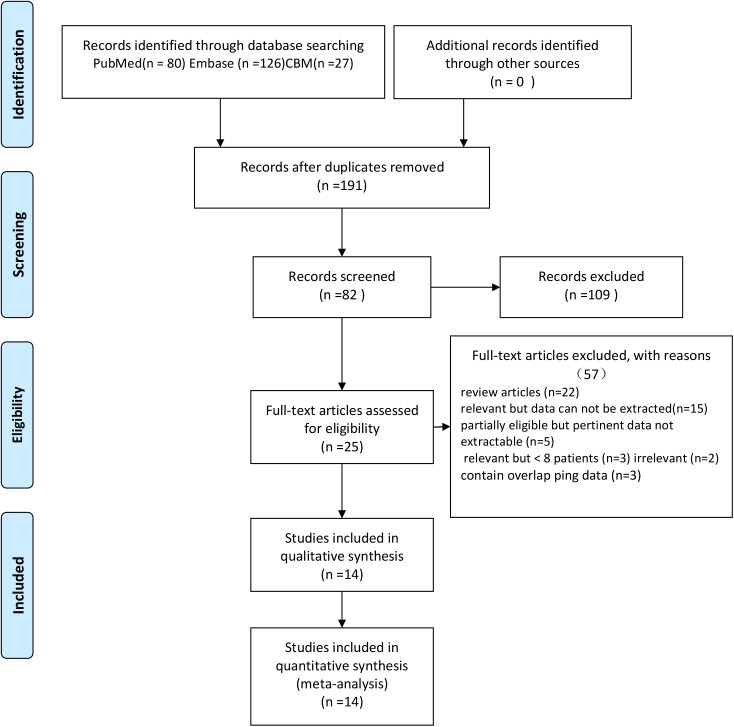
Flow diagram of the study selection process.

A total of 67 articles were screened based on their abstracts and our inclusion/exclusion criteria; 17 of these articles were potentially eligible for further assessment. After a full-text review, the remaining 14 studies evaluating patients with high-grade glioma vs primary central nerve system lymphoma (PCNSL) using MR perfusion met the eligibility criteria for the meta-analysis [9–10,21–24,25–28,29–32]. The study selection flow is displayed in Fig 1. The detailed characteristics of all 14 articles are summarized in Table 1. (More details could be reached in S2 Table)

**Table 1:** Characteristics of studies included in the meta-analysis of PWI for the differential diagnosis of HGGs from PCNSLs.

Author	Year	Country	Study Design	No. of Patients	Age	M/F	Histology	HGG Grading	Reference Standard	MRI	Position of ROI	Analysis Software	Time and Amount (agent)	TYPE of Technique	Parameter	Cutoff
Koji Yamashita	2016	Japan.	R	42	61.21(6–85)	27/23	PCNSL(13);HGG(29)	Ⅳ(29)	path	3T	intra	na	na	IVIM	f max	12.40%
Shanshan Lu	2016	China	R	54	54.67(29–79)	28/26	PCNSL(16);HGG(38)	Ⅳ(38)	path	3T	intra	OmniKinetics	0.1mmol/kg: 4ml/sec	DCE	Ktrans	0.187
															Ve	0.387
Yoon Seong Choi	2016	Korea	R	42	59.7±2.1	18/24	PCNSL(19);HGG(23)	Ⅳ(23)	path	3T	intra	MIPAV	0.1mmol/kg: 3ml/sec	DCE	IAUC30mean	12.2
															IAUC30(90th%)	17.9
															IAUC60(90th%)	40.7
															IAUC90(90th%)	66
Satoshi Nakajima	2015	Japan	R	34	60.87(16–90)	14/17	PCNSL(11);HGG(23)	Ⅳ(23)	path	3T	intra	MIStar	0.1mmol/kg: 3ml/sec	DSC	uncorrect CBV	2.09
Wang Yufang	2015	China	R	31	52.19(22–82)	19/12	PCNSL(11);HGG(20)	Ⅲ(3),Ⅳ(17)	path	3T	intra	Functool	no need	p CASL	m TBF	57.9
															r TBF	141.1
Chong Hyun Suh	2014	Korea	R	60	54.1(25–83)	33/27	PCHSL(19);HGG(41)	Ⅳ(41)	path	3T	intra	Nordic ice(ncbv):Matlab(IVIM)	0.1mmol/kg: 4ml/sec	IVIM	IVIM	0.042
														DSC	n CBV	4.02
P. Kickingereder	2014	Germany	R	71	na	na	PCNSL(11);HGG(60)	Ⅳ(60)	path	3T	intra	Tissue 4D	0.1mmol/kg: 5ml/sec	DCE	Ktrans	0.093
															Kep	0.272
															Ve	0.41
J. Furtner	2014	Austria	P	30	58.7(22–80)	16/14	PCNSL(8);HGG(22)	Ⅳ(22)	path	3T	intra	na	no need	ASL	n VITS	1.41
Z. Xing	2013	China	R	38	50.3	21/27	PCNSL(12);HGG(26)	na	path	3T	intra	Perfusion MR and Mean Curve software	0.1mmol/kg: 5ml/sec	DSC	r CBV	2.56
															r CBF	2.18
															MTT	0.95
															SI	89%
Roh-Eul Yoo	2013	Korea	R	29	51.59(22–82)	na	PCNSL(9);HGG(20)	Ⅲ(3),Ⅳ(17)	path	1.5T	intra	na	no need	ASL	m TBF	45.4
															r TBF	149.7
C.H.Toh	2012	Taiwan	P	35	58.5(22–81)	27/8	PCNSL(15);HGG(20)	Ⅳ(20)	path	3T	intra	Nordic ice	0.1mmol/kg: 4ml/sec	DSC	uncorrect CBV	1.88
															correct CBV1	3.01
															K2	1.2
Koji Yamashita	2012	Japan	R	47	60.64(8–83)	na	PCNSL(12);HGG(35)	Ⅳ(35)	path	3T	intra	IDL	no need	ASL	a TBF	46
															r TBF	1.25
J.H. Ma	2010	Korea	R	40	46(15–73)	33/29	PCNSL(12);HGG(28)	Ⅳ(28)	path	3T	intra and peri	Nordic ice	0.1mmol/kg: 4ml/sec	DSC	HWcel	2.7
															PHPcel	2.7
															MV cel	3.9
															HWpel	1.3
															PHPpel	0.9
															MVpel	1.2
M.A. Weber	2006	Germany	P	45	57±14	43/36	PCNSL(10);HGG(35)	Ⅳ(35)	path	1.5T	intra and peri	Vistar	0.1mmol/kg: 5ml/sec	DSC(ITS-FAIR)	rr CBV	1.4
												na	no need	ASL(Q2TIPS)	rr CBF	1.1
															rr CBF	1.2

R, respective; P, prospective; M, male; F, female; HGG, high grade glioma; PCNSL, primary central nervous lymphoma; path, pathology; na, not available; IVIM, intravoxel incoherent motion; DCE, Dynamic contrast-enhanced; DSC, dynamic susceptibility-weighted, contrast-enhanced; ASL, arterial spin-labeling techniques; intra, intra-tumor; peri, peri-tumor; n CBV, normalized cerebral blood volume; CBV, normalized cerebral blood volume; rr CBF, relative regional cerebral blood flow; a TBF, absolute tumor blood flow; r TBF, relative tumor blood flow; MTT, Maps of mean transit time; SI, signal intensity; HW, histogram width; MV, maximum value; PEL, perienhancing lesion; CEL, contrast-enhancing lesion; PHP, peak height position; IAUC, initial area under the time to signal intensity curve. n VITS, normalized intratumoral signal intensity value.

As shown in Table 1, eleven studies were retrospective, and only three studies were prospective. Among the 14 studies, the number of participants included in each article ranged from 29 to 71, and 598 patients had an appropriate quality of data (according to the data extraction in ‘Materials and Methods’). These 598 patients had a mean age of 55.8, ranging from 6 to 90. The main reference standards used in each study were pathological analyses obtained from biopsy and/or resection. In these 598 patients, there were 178 PCNSLs and 420 HGGs. Six articles evaluated DSC [21,22,24,25,27,32], 5 studies evaluated ASL [10,23,24,28,31], 3 studies evaluated DCE [26,29,30], and 2 studies evaluated IVIM [9,21].

Regarding the strength of the imaging field, 12 studies utilized 3.0T MRI Scanners, and only 2 studies used 1.5T MRI Scanners [24,28].

**Fig 2:**
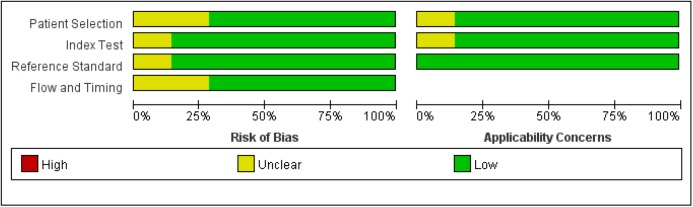
Methodological quality analysis of the 12 eligible studies using QUADAS-2 tool.

The quality test of each study is shown in Fig 2. Most of the studies had a low or unclear risk of bias. Overall, the study quality was eligible.

### 3.2. Quantitative synthesis

#### 3.2.1. Overall analysis

**Fig 3:**
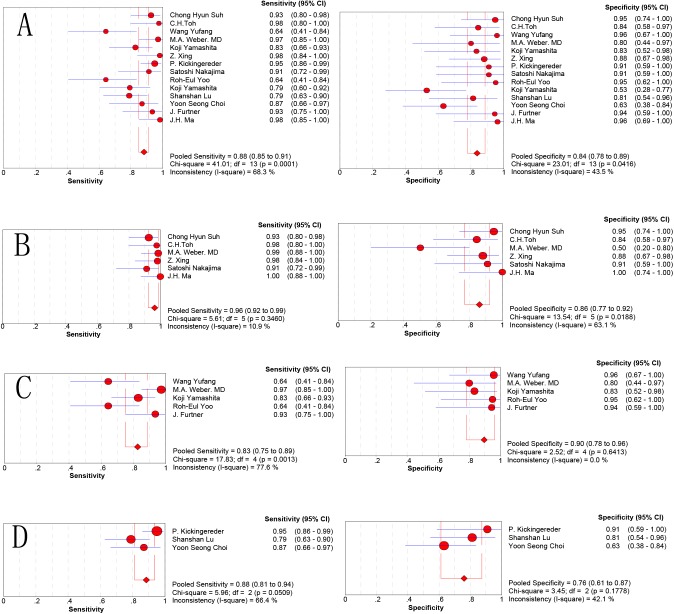
Forest plot showing the sensitivity and specificity of different groups for the differentiation of HGGs from PCNSLs. (A) Overall group; (B) DSC group; (C) ASL group; (D) DCE group.

**Fig 4:**
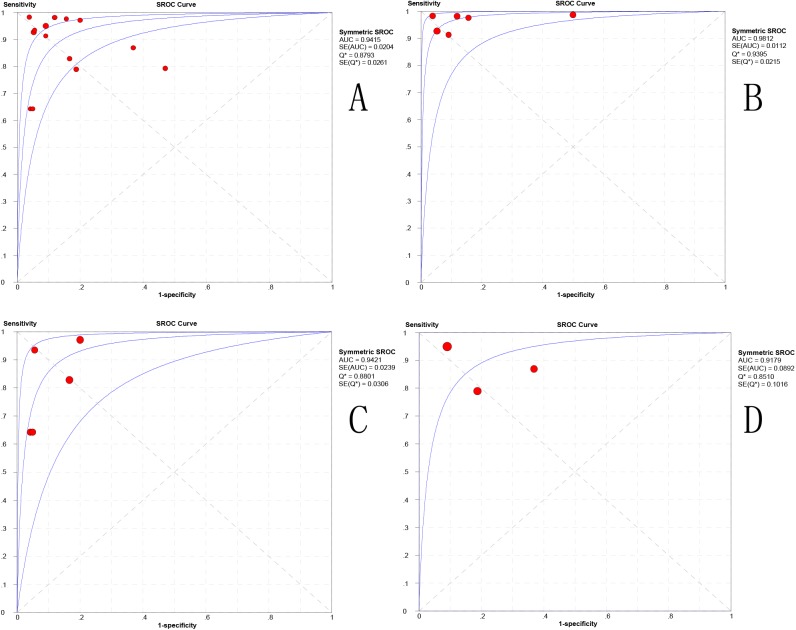
Summary Receiver-Operating Characteristic curve (SROC). (A) Overall group; (B) DSC group; (C) ASL group; (D) DCE group. AUC area under the curve.

The fourteen best-performing parameters from each included study were analyzed to differentiate HGGs from PCNSLs. There was no significant threshold effect, with a Spearman correlation coefficient of -0.077 (P = 0.793). The other values were as follows: pooled SEN: 0.883 (95% CI: 0.848, 0.912); SPE: 0.837(95% CI: 0.777, 0.886); LR+: 5.626 (95% CI: 3.224, 9.818); LR−: 0.145 (95% CI: 0.086, 0.244); and DOR: 53.83 (95% CI: 20.048, 131.43). The forest plots from 14 studies are shown in Fig 3A. The AUC under the SROC was 0.9415 (Fig 4A).

#### 3.2.2. Subgroup analysis

Six studies utilized the technique of DSC to distinguish HGGs from PCNSLs, with the 6 best-performing parameters identified in each study. There was no threshold effect (P = 0.544) or heterogeneity (I2 = 0%) among each study. The pooled SEN/SPE values were 0.963 (95%CI: 0.924, 0.986)/0.861(95% CI: 0.772, 0.925) (Fig 3B). The pooled LR+/LR− was 7.009 (95% CI: 2.516, 19.531) / 0.059 (95% CI: 0.029, 0.122). The pooled DOR was 204.10(95% CI: 62.895, 662.31), and the AUC under the SROC was 0.9812 (Fig 4B).

For ASL, the pooled SEN/SPE values were 0.826 (95%CI: 0.751, 0.886)/0.896(95% CI: 0.781, 0.963), (Fig 3C). The pooled LR+/LR− was 7.454 (95% CI: 3.424, 16.224)/0.218 (95% CI: 0.153, 0.311). The pooled DOR was 47.987(95% CI: 15.765, 146.07), and the AUC under the SROC was 0.9421 (Fig 4C).

For DCE, the pooled SEN/SPE values were 0.884 (95%CI: 0.813, 0.935)/ 0.761(95% CI: 0.612, 0.874) (Fig 3D). The pooled LR+/LR− was 3.942 (95% CI: 2.23, 6.97) /0.173 (95% CI: 0.105, 0.286). The pooled DOR was 21.247 (95% CI: 8.517, 53.007), and the AUC under the SROC was 0.9179 (Fig 4D).

No IVIM parameter was eligible for the subgroup meta-analysis because the minimum required number for each subgroup analysis was three.

### 3.3. Heterogeneity analysis

No severe heterogeneity was found in the pooled analysis in the DSC or ASL groups, but there was severe heterogeneity in the overall and ASL groups.

### 3.4. Publication bias

**Fig 5:**
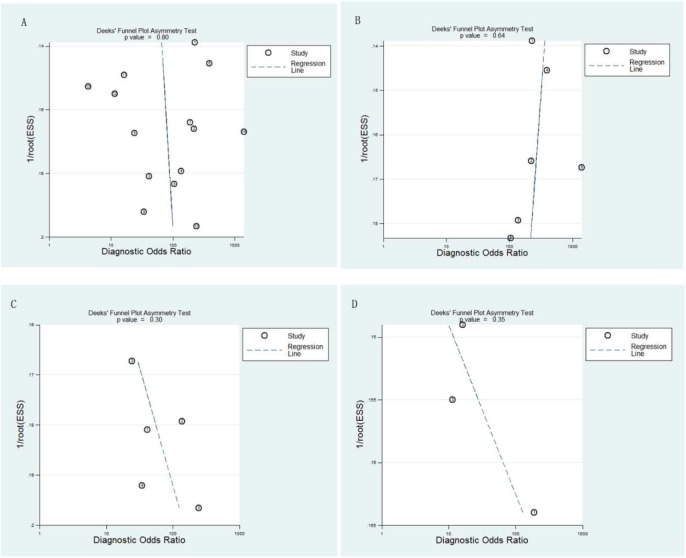
Funnel plot of publication bias. (A) Overall group; (B) DSC group; (C) ASL group; (D) DCE group.

Deek’s funnel plot (Fig 5) asymmetry test showed no significant publication bias for all groups (p = 0.80, p = 0.64, p = 0.3, p = 0.35 for overall, DSC, ASL, DCE group, respectively).

## Discussion

Numerous studies have utilized MR perfusion techniques to discriminate HGGs from PCNSLs [9–10,21–24,25–28,29–32]. In a prior meta-analysis, Liang R[33] only evaluated the role of rCBV values derived from DSC MR imaging. In contrast, we included all of the techniques, including DSC, DCE, ASL and IVIM, to systematically assess the performance of MR perfusion in distinguishing HGGs from PCNSLs.

The degree of malignancy correlates with both the microvascularity and neovascularity of the tumors. A high degree of malignancy increases the microvascularity and neovascularity of tumors and thus increases the tumor blood flow [25–27]. Pathologically, HGG tends to be more malignant than PCNSL, so HCG tends to have a higher level of tumor blood flow and denser vascularity. All of the hemodynamic variables could be measured by using different MR perfusion imaging techniques.

In the results, the AUC for the overall group was 0.9415, which indicated a high diagnostic accuracy of the PWI to distinguish HGGs from PCNSLs. The DOR is a single indicator of test performance that combines the SEN and SPE data into a single number [34]. The pooled DOR for diagnostic accuracy of the overall group was 55.83, which indicated that the use of MR perfusion might be helpful in distinguishing HGGs from PCNSLs. LR+ and LR−are also adopted as ways to assess the diagnostic accuracy of the test because these values appear to be more significant in clinical practice than are the SROC curve and the DOR. A LR >10 or <0.1 always means great and consequential shifts from pre-test to post-test probability and show a good diagnostic accuracy [35]. The value of the LR+ for the overall group was 5.63, which suggests that patients with HGGs were approximately six times more likely to have a positive test than patients with PCNSLs. In contrast, the LR−value was 0.145, which indicates that if the value of the best parameter was lower than the corresponding cut-off value, the probability for this patient to be diagnosed with HGG would be 14.5%, which is not sufficiently low to exclude HGGs. There was evidence of heterogeneity in the overall group, but this heterogeneity was not caused by threshold effect. Therefore, we conducted a meta-regression analysis, which demonstrated that the source of the heterogeneity might come from the MR perfusion imaging technique (p = 0.001). Deek’s funnel plot asymmetry test showed no significant publication bias for the overall group.

For the DSC group, the AUC (0.9812) suggested a high diagnostic accuracy. The pooled DOR for the DSC technique was 204.10, which showed that the DSC technique might be useful in the diagnosis of HGGs. There was no evidence of heterogeneity or publication bias among the 6 relevant studies, which indicated that the results for the DSC technique were statistically credible.

For the ASL group, the AUC (0.9421) also indicated a high diagnostic accuracy. The pooled DOR for diagnostic accuracy of the ASL technique was 47.987, which showed that the DSC technique might also be useful in the diagnosis of HGGs. There was no evidence of heterogeneity or publication bias among the 5 relevant studies, which meant that the results for the ASL technique were statistically credible.

For the DCE group, the AUC (0.9179) also showed a high diagnostic accuracy. The pooled DOR for diagnostic accuracy of the DCE technique was 21.247, which showed that the DSC technique might also be useful in the diagnosis of HGGs. Evidence of heterogeneity was observed for the DCE technique. A meta-regression indicated that the design and strength of MRI might contribute to the heterogeneity because the three studies were all retrospective and used 3T MRI. No publication bias was observed in the DCE group.

The DSC is a widely-used technique in the literature for assessing intracranial mass lesions [36, 37]. The results of the DSC technique in this meta-analysis showed higher diagnostic accuracy (AUC: 0.9812) than the other two techniques (AUC: ASL, 0.9421; DCE, 0.9179), demonstrating that the DSC technique has higher diagnostic accuracy than the ASL and DCE group in distinguishing HGGs from PCNSLs. However, the results of DSC perfusion imaging could be affected by the T2* and T1 effects due to contrast agent leakage. ASL is an emerging MR perfusion imaging technique that requires no extrinsic tracer or radiation exposure, which is an advantage of ASL over other perfusion imaging techniques. ASL also showed high accuracy in clinical applications [10,38–41]. Furthermore, several studies have displayed the successful application of DCE-MR imaging for the quantitative evaluation of vascular permeability parameters, although its limitations affect its clinical use [42–43].

The Youden index (sensitivity+specificity-1), a combinatory index of sensitivity and specificity at a cut-point, summarizes the discriminatory accuracy of a diagnostic test [44]. Based on the overall study analysis, the Youden index for the differentiation of HGGs from PCNSLs was higher for the DSC technique (0.824) than for the ASL technique (0.722) or the DCE technique (0.645). Considering this diagnostic performance, the DSC technique might be an optimal index for distinguishing HGGs from PCNSLs. Additionally, the DSC technique holds the best sensitivity (0.963) compared with the other two techniques (ASL/DCE: 0.826/0.884), whereas the ASL technique displayed the best specificity (ASL/DSC/DCE:0.896/0.861/0.761) in the discrimination.

However, given the limited data, a further subgroup analysis for the DSC technique is needed to find the optimal parameter and its cut-off value in differentiating HGGs from PCNSLs.

### Limitations

There were several limitations in our meta-analysis, although the MR perfusion showed a high diagnostic accuracy.

First, most of the included studies adopted multiple and different parameters to evaluate the performance of the MR perfusion; therefore, the optimal parameter and threshold value remain difficult to identify due to the highly variable proposed cutoff values, and the conclusion drawn from each study is potentially valuable only as a general guide. Further evaluation and standardization of the techniques and post-processing methods are needed before the techniques can be extensively clinically used. Second, we included patients who had been diagnosed with WHO grade III glioma, whereas the majority of the patients included were grade IV. Thus, the different tumor biology and angiogenesis might have impact on the results. Third, there was evidence of heterogeneity among the overall and DCE groups. Factors such as different field strengths, types of techniques and post-processing methods might have contributed to this heterogeneity. Although heterogeneity was not found in the DSC and ASL groups, there were differences among the studies, such as age, gender, study designs, parameters and MR devices.

## Conclusions

This meta-analysis revealed a high level of accuracy of the PWI to distinguish HGGs from PCNSLs. Among the MR perfusion imaging techniques, DSC might be an optimal index for distinguishing HGGs from PCNSLs. Furthermore, the DSC technique showed the best sensitivity. and the ASL technique displayed the best specificity. However, the variability of optimal thresholds from the included studies suggests that further evaluation and standardization are needed before the methods can be extensively clinically used.

## Supporting information

S1 Table PRISMA-2009-Checklist. (DOC)

S2 Table Detailed description of the studies included. (DOCX)
