# Supplementary material for: The performance of MR perfusion-weighted imaging for the differentiation of high-grade glioma from primary central nervous system lymphoma: A systematic review and meta-analysis
Source: PLoS One. 2017 Mar 16;12(3):e0173430. doi: 10.1371/journal.pone.0173430 (PMC5354292; doi:10.1371/journal.pone.0173430)
Supplement: S2 Table — (DOCX) [file pone.0173430.s002.docx]

| TABLE 1 Characteristics of studies included in the meta-analysis of PWI for the differential diagnosis of HGGs from PCNSLs. | | | | | | | | | | | | | | | | | | | | | |
| --- | --- | --- | --- | --- | --- | --- | --- | --- | --- | --- | --- | --- | --- | --- | --- | --- | --- | --- | --- | --- | --- |
| Author | Year | Country | Study design | No.of patients included | Age | M/F | Histology | HGG grading | Reference standard | MRI | Position of ROI | analysis software | time and ammount(agent) | TYPE of technique | parameter | cutoff | mean value | TP | FP | FN | TN |
| Koji Yamashita | 2016 | Japan. | R | 42 | 61.21(6-85) | 27/23 | PCNSL(13);HGG(29) | Ⅳ(29) | path | 3T | intra | na | na | IVIM | f max | 12.40% | HGG:16.4%±6.26%; PCNSL:11.8%±4.26% | 23 | 8 | 6 | 9 |
| Shanshan Lu | 2016 | China | R | 54 | 54.67(29-79) | 28/26 | PCNSL(16);HGG(38) | Ⅳ(38) | path | 3T | intra | OmniKinetics | 0.1mmol/kg ；4ml/sec | DCE | Ktrans | 0.187 | HGG:0.149±0.088; PCNSL:0.289±0.124 | 30 | 3 | 8 | 13 |
|  |  |  |  |  |  |  |  |  |  |  |  |  |  |  | Ve | 0.387 | HGG:0.366±0.280; PCNSL:0.556±0.223 | 25 | 3 | 13 | 13 |
| Yoon Seong Choi | 2016 | Korea | R | 42 | 59.7±2.1 | 18/24 | PCNSL(19);HGG(23) | Ⅳ(23) | path | 3T | intra | MIPAV | 0.1mmol/kg ；3ml/sec | DCE | IAUC30mean | 12.2 | HGG:13.2±1.9; PCNSL:11.8±1.9 | 15 | 4 | 8 | 15 |
|  |  |  |  |  |  |  |  |  |  |  |  |  |  |  | IAUC30(90th%) | 17.9 | HGG: 18.6±2.4; PCNSL:16.1±2.0; | 20 | 7 | 3 | 12 |
|  |  |  |  |  |  |  |  |  |  |  |  |  |  |  | IAUC60(90th%) | 40.7 | HGG:42.8±4.7; PCNSL:38.9±4.4 | 16 | 6 | 7 | 13 |
|  |  |  |  |  |  |  |  |  |  |  |  |  |  |  | IAUC90(90th%) | 66 | HGG:68.3±6.3; PCNSL:63.9±6.5; | 16 | 6 | 7 | 13 |
| Satoshi Nakajima | 2015 | Japan | R | 34 | 60.87(16-90) | 14/17 | PCNSL(11);HGG(23) | Ⅳ(23) | path | 3T | intra | MIStar | 0.1mmol/kg ；3ml/sec | DSC | uncorrect CBV | 2.09 | HGG:4.99±2.89; PCNSL:1.57±0.56 | 21 | 1 | 2 | 10 |
| Wang Yufang | 2015 | China | R | 31 | 52.19(22-82) | 19/12 | PCNSL(11);HGG(20) | Ⅲ(3),Ⅳ(17) | path | 3T | intra | Functool | no need | p CASL | m TBF | 57.9 | HGG:92.1±34.7; PCNSL:53.6±30.5 | 18 | 5 | 2 | 6 |
|  |  |  |  |  |  |  |  |  |  |  |  |  |  |  | r TBF | 141.1 | HGG:182.3±69.5; PCNSL:92.5±44.9 | 13 | 0 | 7 | 11 |
| Chong Hyun Suh | 2014 | Korea | P | 60 | 54.1(25-83) | 33/27 | PCHSL(19);HGG（41） | Ⅳ(41) | path | 3T | intra | Nordic ice（ncbv）；Matlab（IVIM） | 0.1mmol/kg ；4ml/sec | IVIM | IVIM | 0.042 | glio：0.101±0.016；PCNSL：0.021±0.010 | 39 | 2 | 2 | 17 |
|  |  |  |  |  |  |  |  |  |  |  |  |  |  | DSC | n CBV | 4.02 | glio:7.05±0.98；PCNSL:3.02±1.09 | 38 | 1 | 3 | 18 |
| P. Kickingereder | 2014 | Germany | R | 71 | na | na | PCNSL(11);HGG(60) | Ⅳ(60) | path | 3T | intra | Tissue 4D | 0.1mmol/kg ；5ml/sec | DCE | Ktrans | 0.093 | HGG:0.064±0.021; PCNSL:0.145±0.057 | 57 | 1 | 3 | 10 |
|  |  |  |  |  |  |  |  |  |  |  |  |  |  |  | Kep | 0.272 | HGG:0.230±0.058; PCNSL:0.396±0.088 | 47 | 1 | 10 | 13 |
|  |  |  |  |  |  |  |  |  |  |  |  |  |  |  | Ve | 0.41 | HGG:0.319±0.107; PCNSL:0.434±0.165 | 46 | 4 | 7 | 14 |
| J. Furtner | 2014 | Austria | P | 30 | 58.7(22-80) | 16/14 | PCNSL(8);HGG(22) | Ⅳ(22) | path | 3T | intra | na | no need | ASL | n VITS | 1.41 | HGG:1.3-5.24; PCNSL:0.95±1.37; | 21 | 0 | 1 | 8 |
| Z. Xing | 2013 | China | R | 38 | 50.3 | 21/27 | PCNSL(12);HGG(26) | NA | path | 3T | intra | Perfusion MR and Mean Curve software | 0.1mmol/kg ；5ml/sec | DSC | r CBV | 2.56 | HGG:5.05±1.65; PCNSL:1.69±0.78 | 25 | 2 | 1 | 18 |
|  |  |  |  |  |  |  |  |  |  |  |  |  |  |  | r CBF | 2.18 | HGG:5.09±2.45; PCNSL:2.50±1.41 | 26 | 8 | 0 | 12 |
|  |  |  |  |  |  |  |  |  |  |  |  |  |  |  | MTT | 0.95 | HGG:1.16±0.21; PCNSL:0.69±0.20 | 23 | 2 | 3 | 18 |
|  |  |  |  |  |  |  |  |  |  |  |  |  |  |  | SI | 89% | HGG:68±21%; PCNSL:137±59% | 26 | 2 | 0 | 18 |
| Roh-Eul Yoo | 2013 | Korea | R | 29 | 51.59(22-82) | na | PCNSL(9);HGG(20) | Ⅲ(3),Ⅳ(17) | path | 1.5T | intra | na | no need | ASL | m TBF | 45.4 | HGG:92.1±34.7; PCNSL:53.6±30.5 | 20 | 4 | 0 | 5 |
|  |  |  |  |  |  |  |  |  |  |  |  |  |  |  | r TBF | 149.7 | HGG:182.3±69.5; PCNSL:92.5±44.9 | 13 | 0 | 7 | 9 |
| C.H.Toh | 2012 | Taiwan | P | 35 | 58.5(22-81) | 27/8 | PCNSL(15);HGG(20) | Ⅳ(20) | path | 3T | intra | Nordic ice | 0.1mmol/kg ；4ml/sec | DSC | uncorrect CBV | 1.88 | HGG:5.01±2.01; PCNSL:1.16±0.66 | 20 | 2 | 0 | 13 |
|  |  |  |  |  |  |  |  |  |  |  |  |  |  |  | correct CBV1 | 3.01 | HGG:5.47±2.05; PCNSL:2.28±0.60 | 18 | 1 | 2 | 14 |
|  |  |  |  |  |  |  |  |  |  |  |  |  |  |  | K2 | 1.2 | HGG:0.79±0.68; PCNSL:1.88±1.11 | 15 | 3 | 5 | 12 |
| Koji Yamashita | 2012 | Japan | R | 47 | 60.64(8-83) | na | PCNSL(12);HGG(35) | Ⅳ(35) | path | 3T | intra | IDL | no need | ASL | a TBF | 46 | HGG:91.6±56; PCNSL:37.3±10.5 | 29 | 2 | 6 | 10 |
|  |  |  |  |  |  |  |  |  |  |  |  |  |  |  | r TBF | 1.25 | HGG:2.61±1.61; PCNSL:1.24±0.37 | 29 | 5 | 6 | 7 |
| J.H. Ma | 2010 | Korea | R | 40 | 46(15-73) | 33/29 | PCNSL(12);HGG(28) | Ⅳ(28) | path | 3T | intra and peri | Nordic ice | 0.1mmol/kg ；4ml/sec | DSC | HWcel | 2.7 | HGG,6.01±1.15;PCNSL 1.93± 0.79 | 28 | 0 | 0 | 12 |
|  |  |  |  |  |  |  |  |  |  |  |  |  |  |  | PHPcel | 2.7 | HGG,4.79±1.31;PCNSL 2.08± 0.54 | 27 | 0 | 1 | 12 |
|  |  |  |  |  |  |  |  |  |  |  |  |  |  |  | MV cel | 3.9 | HGG,7.43±1.28;PCNSL 2.83±0.83 | 28 | 0 | 0 | 12 |
|  |  |  |  |  |  |  |  |  |  |  |  |  |  |  | HWpel | 1.3 | HGG 1.60±0.22; PCNSL 0.71± 0.42 | 24 | 0 | 4 | 12 |
|  |  |  |  |  |  |  |  |  |  |  |  |  |  |  | PHPpel | 0.9 | HGG,1.42±0.28;PCNSL 0.91±0.37 | 27 | 1 | 1 | 11 |
|  |  |  |  |  |  |  |  |  |  |  |  |  |  |  | MVpel | 1.2 | HGG 1.90±0.26; PCNSL 1.27± 0.34 | 26 | 0 | 2 | 12 |
| M.A. Weber | 2006 | Germany | P | 45 | 57±14 | 43/36 | PCNSL(10);HGG(35) | Ⅳ(35) | path | 1.5T | intra and peri | Vistar | no need | ASL | rrCBF(ITSFAIR) | 1.4 | na | 35 | 5 | 0 | 5 |
|  |  |  |  |  |  |  |  |  |  |  |  | na | 0.1mmol/kg ；5ml/sec |  | rr CBF(Q2TIPS) | 1.1 |  | 31 | 2 | 4 | 8 |
|  |  |  |  |  |  |  |  |  |  |  |  |  |  | DSC | rr CBV | 1.2 |  | 34 | 2 | 1 | 8 |
| R, respective; P, prospective; M, male; F, female; HGG, high grade glioma; PCNSL, primary central nervous lymphoma; path, pathology; na, not available; IVIM, intravoxel incoherent motion; DCE, Dynamic contrast-enhanced; DSC, dynamic susceptibility-weighted, contrast-enhanced; ASL, arterial spin-labeling techniques; intra, intra-tumor; peri, peri-tumor; n CBV, normalized cerebral blood volume; CBV, normalized cerebral blood volume; rr CBF, relative regional cerebral blood flow; a TBF, absolute tumor blood flow; r TBF, relative tumor blood flow; TP, true positive; FP, false positive; TN, true negative; FN, false negative; MTT, Maps of mean transit time; SI, signal intensity; HW, histogram width; MV, maximum value; PEL, perienhancing lesion; CEL, contrast-enhancing lesion; PHP, peak height position; IAUC, initial area under the time to signal intensity curve. n VITS, normalized intratumoral signal intensity value. | | | | | | | | | | | | | | | | | | | | | |
